# Mechanism of bubbles formation and anomalous phase separation in the CoNiP system

**DOI:** 10.1038/s41598-023-33146-7

**Published:** 2023-04-10

**Authors:** Maria I. Panasyuk, Tatiana I. Zubar, Tatsiana I. Usovich, Daria I. Tishkevich, Oleg D. Kanafyev, Vladimir A. Fedkin, Anna N. Kotelnikova, Sergei V. Trukhanov, Dominik Michels, Dmitry Lyakhov, Tatiana N. Vershinina, Valery M. Fedosyuk, Alex V. Trukhanov

**Affiliations:** 1 Laboratory of Magnetic Films Physics, Scientific-Practical Materials Research Centre of National Academy of Sciences of Belarus, 220072 Minsk, Belarus; 2 Smart Sensors Laboratory, Department of Electronic Materials Technology, National University of Science and Technology MISiS, Moscow, Russia 119049; 3 Computer, Electrical and Mathematical Science and Engineering Division, 4700 King Abdullah University of Science and Technology, Thuwal, 23955-6900 Kingdom of Saudi Arabia; 4 Joint Institute for Nuclear Research, Dubna, Russia 141980; 5 Dubna State University, Dubna, Russia 141980

**Keywords:** Chemistry, Materials science, Physics

## Abstract

This study announces the anomalous phase separation in CoNiP alloy electroplating. The observed phenomenon of the formation of magnetic bubbles was described for the first time for this triple CoNiP system. This study briefly covers all stages of magnetic bubble formation, starting from the formation of an amorphous phosphor-rich sublayer, followed by nucleation centers, and finally cobalt-rich bubbles. An explanation for the anomalous mechanism of bubble formation was found in the effects of additives and the phenomena of depolarization and superpolarization.

## Introduction

Solving the problems of creating new and improving existing magnetic materials is an important task for researchers. Due to the widespread use of magnetic materials in microelectronics and various fields of modern technology, the development of materials and devices based on them that have increasingly high requirements is an extremely relevant direction^1–10^.

The formation and production of magnetic field sensors, magnetic recording heads, electromagnetic radiation shields, laser bodies, instruments, and aviation and space technology devices often require the unique physical properties of metals and alloys. It is known that the main properties of coatings are determined by their composition^11^. Therefore, it is extremely important to obtain the composition of coatings for certain applications and control their properties with various obtaining methods^12,13^. Also, when studying magnetic materials, new questions arise related to the thickness of coatings, such as the specific structure and growth mechanism^14–20^. Based on the above, the problem of changing patterns in the properties of coatings resulting from their composition, thickness, and production parameters arises.

There are many different methods of obtaining coatings, such as thermal evaporation, cathode sputtering, chemical and electrolytic deposition, etc. However, we believe that electrochemical deposition is the most suitable method for the manufacture of magnetic coatings. This method is widely used due to its simplicity and low cost compared to other methods of obtaining magnetic coatings. An important advantage is the possibility of obtaining precipitation with predetermined properties by changing the deposition parameters. To obtain the properties of interest to us, it is possible to vary the current density, temperature, pH value, electrolyte composition, and concentration of components in it over a wide range. Taking into consideration the variety of different parameter combinations, a wide area opens up for investigating the effect of deposition parameters on the properties of the resulting sediment. Due to strict growth control, suitable magnetic properties can be maintained in thick coatings. In addition, the method is compatible with traditional semiconductor manufacturing technologies used in microelectronics, which makes the electrochemical deposition method suitable for industrial production of magnetic films.

Cobalt-based alloys are widely used in industry^21^. Such a wide distribution is explained by the properties of this metal. Cobalt is a metal with high endurance, hardness, and heat resistance^22^. In addition, due to its heat resistance, cobalt can be used in the aviation and space industries at high temperatures, at which nickel loses strength.

The study of cobalt-based triple alloys is extremely relevant. Triple alloys are widely used in industry due to the possibility of wide variations in their properties by changing the composition. Numerous studies have been conducted on the development of triple CoNiP coatings due to their potential application in high-density recording and microelectromechanical systems^23–26^. In the research area of electrochemically deposited CoNiP alloys, it is of interest to clarify the mechanism of coating formation, the action of additives, and changes in the concentrations of electrolyte components. In some cases of CoNiP coating deposition, it is not always possible to use the common mechanisms of electrochemical alloy deposition to describe the process of their formation. Therefore, research on the formation conditions of the coating during electrolysis and their effect on its structure can be useful for determining the mechanism of electrochemical deposition of the CoNiP alloy.

This article describes the preparation of CoNiP coatings with spherical formations on the surface. An anomalous cobalt release was revealed in the active centers. The formation of spherical deposits on the surface was explained by the phenomenon of cobalt depolarization.

## Materials and methods

Galvanostatic electrodeposition of Co–Ni-P alloy thin films from an aqueous electrolytic solution was used. The electrolyte for the deposition of CoNiP films was an amino-cloride-based solution containing 0,21 M CoSO_4_, 0,20 M NiSO_4_, 0,15 M NaH_2_PO_2_, 0,40 M H_3_BO_3_, 0,70 M NH_4_Cl, and 0,005 M saccharin. The copper substrates are first degreased with Viennese lime, followed by immersion in an (NH_4_)_2_S_2_O_8_ + H_2_SO_4_ bath for 60 s to etch. The pH of the solution was adjusted with H_2_SO_4_, and the solution was maintained at 80 °C during deposition without any stirring. The experiment was conducted at 10 mA·cm^–2^; nickel sheet was used as a soluble anode. Nickel-sulfate and cobalt-sulfate are used for the metal-ions source. Boric acid was added as a pH buffer. Sodium hypophosphite supplies the ions for the cathode and anode reactions. To reduce deposit stress, NH_4_Cl was added as a supporting electrolyte with saccharin. The depositions were carried out at various deposition times ranging from 1 to 60 min (Table 1).

**Table 1:** Deposition time.

Sample name	Deposition time, minute
CoNiP1	1
CoNiP10	10
CoNiP20	20
CoNiP30	30
CoNiP60	60

**Figure 1:**
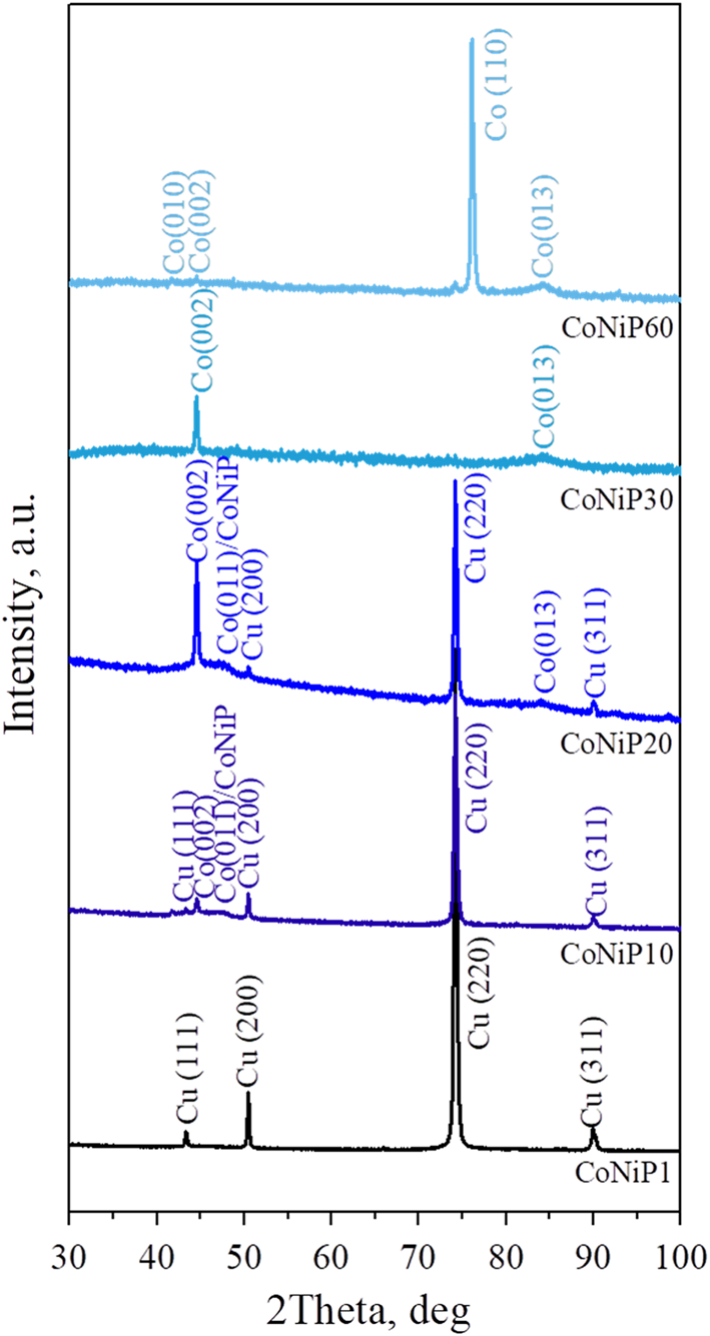
Diffractograms of coatings applied during 1, 10, 20, 30, and 60 min.

Figure 1 shows X-ray images of coatings. Studies carried out by the X-ray method in the Bregg-Brentano focusing on Cu-Ka radiation (Fig. 1) showed that after coating for 1 min (CoNiP1), peaks of the copper substrate are detected, and the ratio of peak intensities corresponds to the rolling texture. As the coating time increases to 10 min (CoNiP10) or more (CoNiP15–CoNiP60), peaks corresponding to a cobalt-based solid solution appear. In general, cobalt can be formed with both HCP (hexagonal close packed) and FCC (face centered cubic) lattices. But due to the fact that the coatings have a pronounced crystallographic texture, it is quite difficult to separate them. Moreover, the elemental composition varies, and consequently, the parameters of the crystal lattice can also change. We can say for sure that there is a HCP lattice only for the "60-min" state (CoNiP60), when a weakly expressed peak of Co (010) appears. Therefore, further arguments are conducted from the point of view of the HCP lattice.

It is worth noting that the cobalt-based solid solution (in which nickel can replace cobalt atoms) has a predominant crystallographic orientation and that its character changes as the coating time increases. If the peak of Co(002) was the most intense in the initial stages (CoNiP10 and CoNiP20), its intensity gradually decreased (CoNiP30), then almost completely disappeared (CoNiP60), and the peak of Co(110) became the most intense.

Regarding the CoNiP phase, if it is in a highly dispersed state (X-ray amorphous), then a wide peak at ~ 45° will be present on the diffractogram. And it really is on the diffractograms of samples with coatings applied within 10 and 20 min (CoNiP10 and CoNiP20). The same conditions correspond to a higher concentration of phosphorus compared to coatings applied for 30 and 60 min (CoNiP 30 and CoNiP 60).

However, at ~ 45°, there may be a peak from a cobalt HCP-based solid solution. Either there is an overlap of peaks from two phases, or the peaks from a cobalt-based solid solution differ in width, and this may be evidence that the crystallites have a shape very different from spherical. If we pay attention to the diffractograms of samples with 30 (CoNiP30) and 60 (CoNiP60) minutes of coating application, it can be noted that a wide peak appears at ~ 85°, which can also be attributed to reflection from the (013) HCP-Co plane.

If we start from the fact that all peaks belong to a cobalt-based solid solution, then the difference in the width of the peaks can be explained as follows: if a particle has a dedicated crystallographic plane with the lowest surface energy, it will grow in a faceted form, including needles or scales. And thus, the particle will have different sizes in different directions, and on diffractograms, we detect peaks from the same phase of different widths. In the case of doping, a part of the atoms in the crystal lattice is replaced, but the substitution is not chaotic; as a rule, there is a predominant substitution in individual nodes. This leads to the fact that the surface energy of the crystallographic planes can change and, consequently, the shape of the particles can vary.

**Figure 2:**
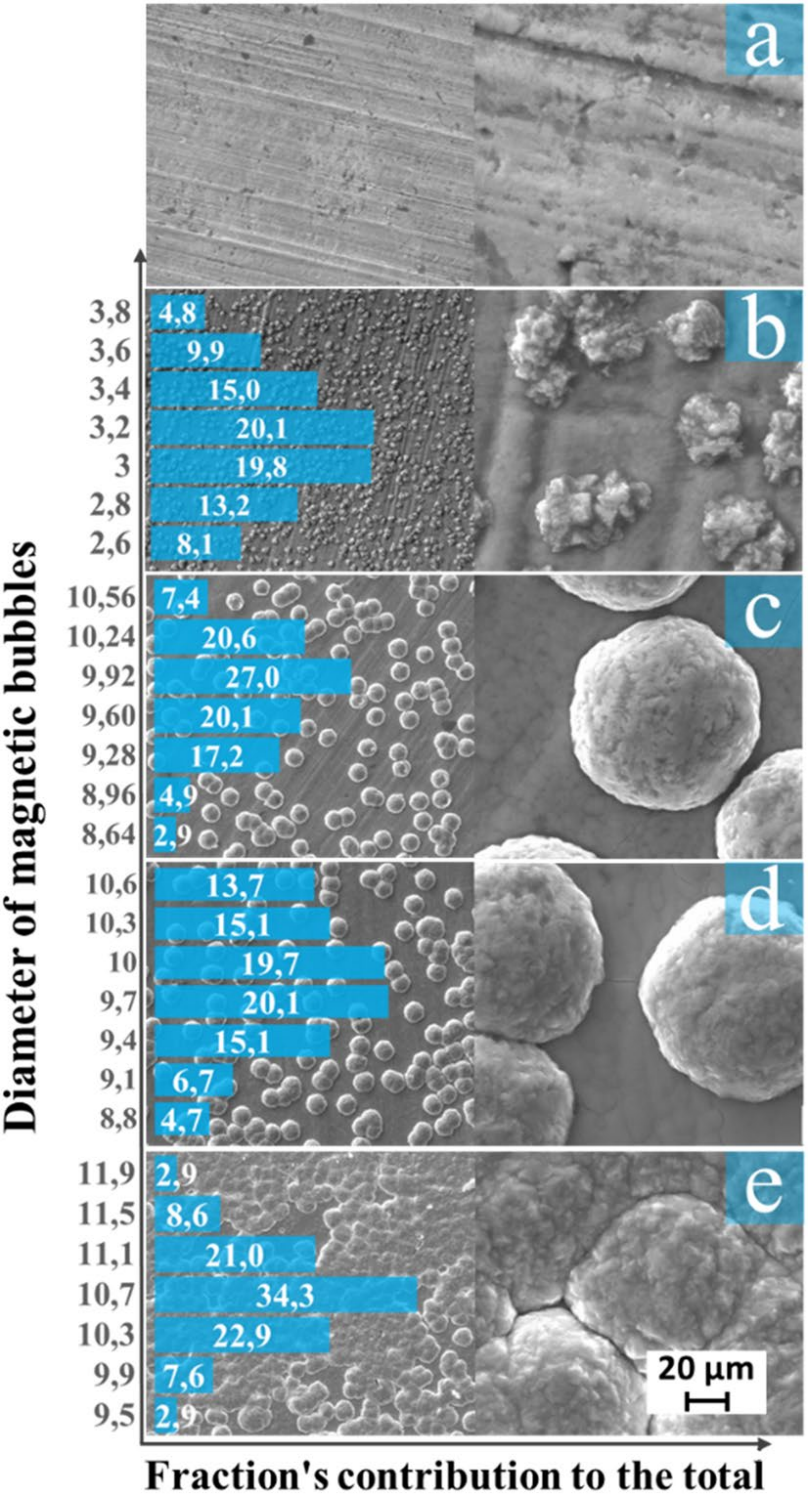
SEM images of the CoNiP films obtained with different deposition times and agglomeratesize distributions (**a**) CoNiP1; (**b**) CoNiP10; (**c**) CoNiP20; (**d**) CoNiP30; (**e**) CoNiP60.

The structure of the obtained coatings is shown in the SEM images (Fig. 2).

During deposition, agglomerates are formed on the surface of the coating. At the initial deposition time, irregularly shaped agglomerates predominate over the entire surface of the coating (Fig. 2b). The size of these formations varies from 2.6 to 4.4 µm. Most agglomerates have a diameter of 3.2–3.8 µm. The morphology of the particles obtained with a longer deposition time is also shown in Fig. 2. As the deposition time increases, the shape of the particles gradually improves. The particles on the surface of the CoNiP20 and CoNiP30 samples are smooth agglomerates of a spherical shape. It is also observed that the number of separately located agglomerates on the sample surfaces is small. On most of the sample surfaces, the particles form chains or islands of agglomerates. With rising time, the number of particles on the sample surfaces increases, and after 60 min of deposition, the agglomerates coalesce with each other and form a new coating layer. The size of these particles changes slightly with increasing time. All spherical sediments are similar in diameter. Their diameter is 9–11 µm and does not depend on the change in deposition time. The number of agglomerates with a diameter of less than 9 or more than 11 µm is insignificant.

**Figure 3:**
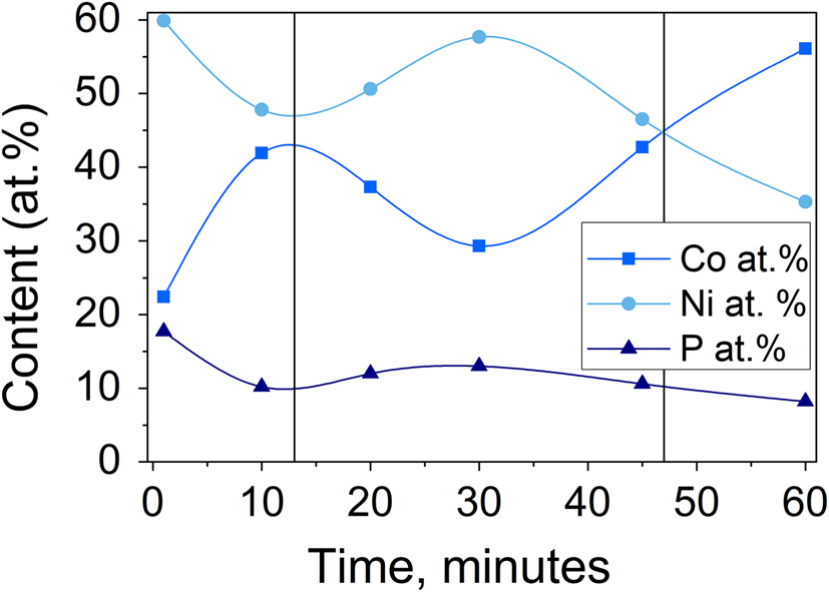
Chemical composition of CoNiP coatings.

The content of Co, Ni, and P in CoNiP coatings was measured by energy dispersion analysis X-ray spectroscopy. Figure 3 shows the dependence of the coating composition on the electrodeposition time. Three areas can be distinguished on the graph. In the first area, a high content of nickel and phosphorus is observed, which decreases with increasing deposition time. In the second area, on the contrary, there is an increase in the nickel content and a decrease in the cobalt content. The phosphorus content increases slightly. In the third area, a significant increase in the cobalt content is observed, and the nickel and phosphorus contents decrease.

**Figure 4:**
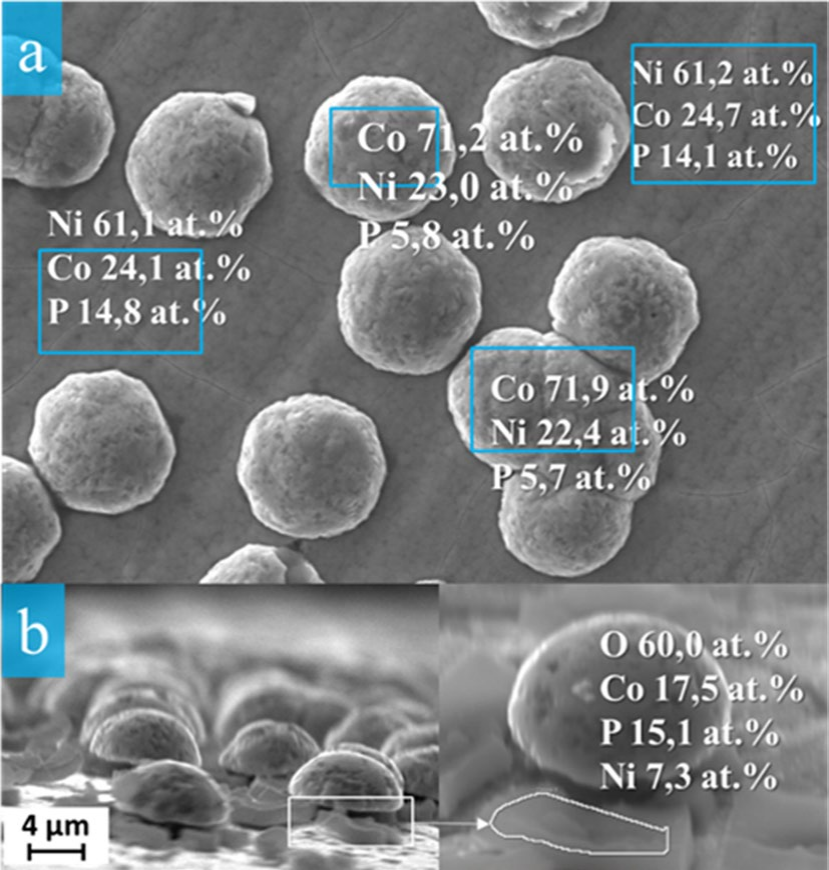
The microstructure and composition of individual sections of the CoNiP20 coatings (**a**) as well as an agglomerate side view (**b**).

Figure 4b demonstrates a SEM image of the CoNiP20 sample`s end face.

It can be seen that some of the formations in the scales form are located under the agglomerates. Agglomerates begin to form on these formations. The composition of individual sections of the CoNiP20 coating surface was also considered. As can be seen from Fig. 4, the smooth coating and agglomerates have a completely different composition. The coating has a high content of nickel and phosphorus, while the agglomerates have a high content of cobalt.

When studying the side view of the surface, it was revealed that the scale formations contain a large amount of phosphorus and oxygen.

A thin coating of NiCoP alloy with a relatively high phosphorus content is formed on the surface in one minute of deposition. At the moment, active centers are beginning to form on the surface of the thin NiCoP coating. During working time, the electrolyte degrades and phosphorus-containing complexes diffuse and adsorb onto the coating surface. This confirms the composition of the scale formations (Fig. 4).

**Figure 5:**
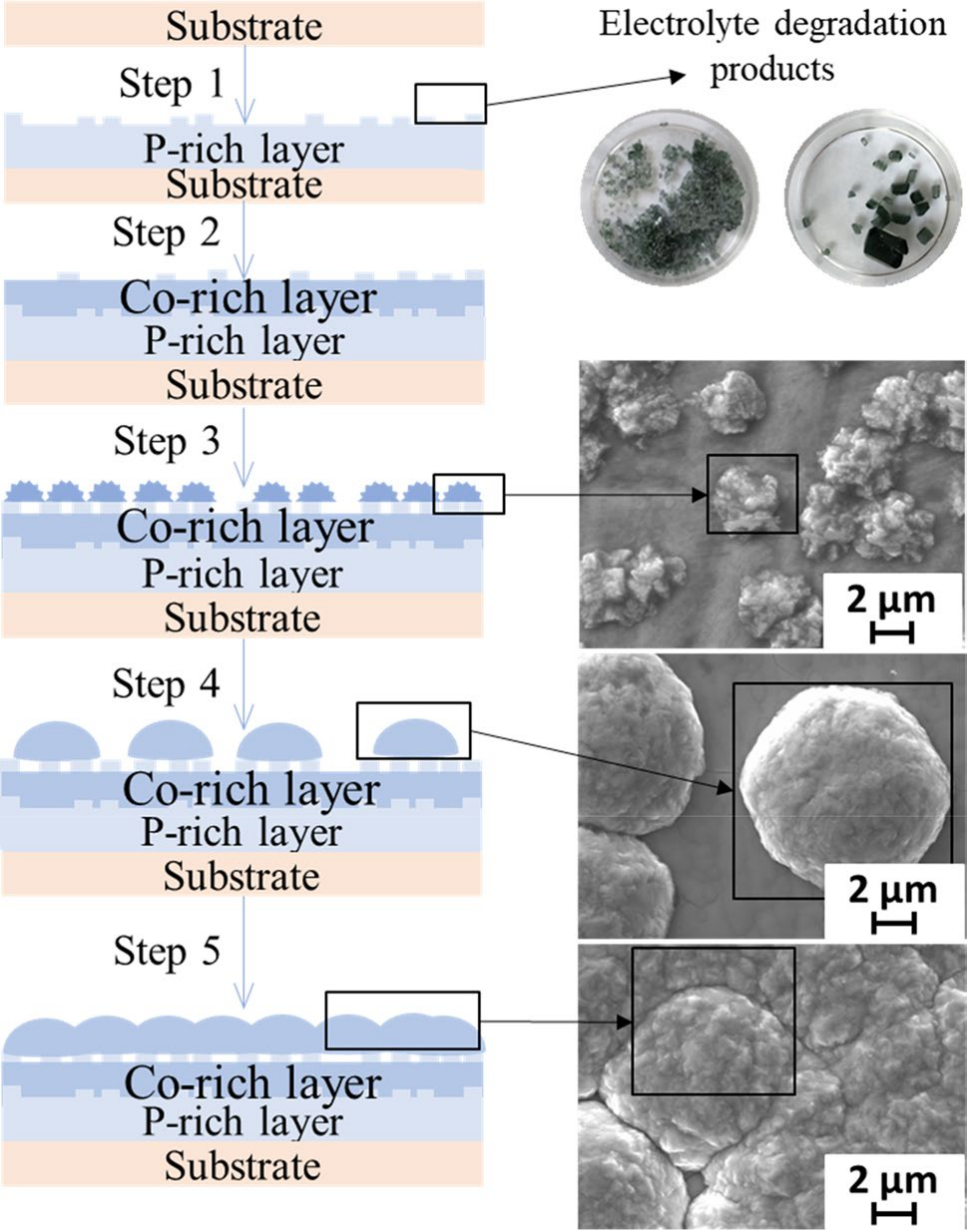
A schematic view of the CoNiP coating growth mechanism.

After 10 min of deposition, the cobalt content of the coating increases, but with an increase in the deposition time, its content in the coating decreases. This can be explained by the fact that cobalt begins to precipitate mainly on active centers, which is confirmed by its high content in spherical agglomerates (Fig. 4). A sharp increase in the cobalt content is observed after 60 min of deposition, when spherical formations coalesce together and form a new continuous coating. Thus, there is an increase in coating by the Stransky—Krystanov mechanism (the mechanism of layer-plus-island growth). Schematically, the mechanism of coating growth is shown in Fig. 5.

The predominant deposition of cobalt on the active centers can be explained by two reasons. Such an effect on the coating can be provided by organic additives (in our case, saccharin). The effect of saccharin on the surface structure is described in a number of articles^27–29^. It can be assumed that an ion-colloidal complex is formed under the influence of the additive. The discharge of this complex at the cathode is difficult compared to the discharge of a conventional hydrated ion. Additives can be adsorbed on the cathode surface, causing an increase in the polarization of the electrode. Adsorption layers have a selective effect on ions of various metals. Thus, the discharge potentials of metals can vary. One of the components can be separated with depolarization (facilitating the separation process) and overpolarization of the other component (inhibiting the separation process).

There is a phenomenon of metal separation with chemical polarization. Numerous studies have been conducted on the electrocrystallization of Co Ni and Fe Ni alloys, which have shown that the more electronegative component of the alloy is deposited with significant de-polarization^30–32^. Conversely, a more electropositive component, which usually precipitates at a higher rate, has superpolarization. Thus, an abnormal increase in the recovery rate of more electronegative cobalt and a simultaneous decrease in the recovery rate of more electropositive nickel are observed.

## Conclusion

The preparation of cobalt spherical particles on copper surfaces using galvanostatic electrodeposition is reported. The cobalt particles are characterized using SEM, XRD, and EDAX. The mechanism of dendritic growth is discussed using the Stransky—Krystanov model. This study covers five stages of magnetic bubble formation: the formation of an amorphous phosphor-rich sublayer (17,7 at.% P); the formation of nucleation centers; the formation of irregularly shaped agglomerates with a diameter of 3.2–3.8 µm; changing the shape of agglomerates from irregular to smooth spherical and increasing their size to 9–11 µm; and finally, the coalescence of agglomerates with each other and the formation of a new coating layer. An explanation for the anomalous mechanisms of bubble formation and anomalous phase separation in the CoNiP system was found in the effect of additives and the phenomena of depolarization and superpolarization.

## Data Availability

Data available upon request to the author Maria I. Panasyuk.
